# Impact of Psychological Violence on Pregnancy Outcomes in a Prospective Study

**Published:** 2014

**Authors:** Fatemeh Abdollahi, Farideh Rezaie Abhari, Jamshid Yazdani Charati, Samad Rouhani

**Affiliations:** 1 Department of Public Health, School of Health, Mazandaran University of Medical Sciences, Sari, Iran.; 2 Department of Midwifery, School of Nursing and Midwifery, Mazandaran University of Medical Sciences, Sari.; 3 Psychiatry and Behavioral Research Center, Addiction Institute, Department of Statistics, School of Health, Mazandaran University of Medical Sciences, Sari, Iran

**Keywords:** Outcomes, Pregnancy, Psychological, Violence

## Abstract

**Objective:** Violence during pregnancy has been associated with adverse pregnancy outcomes. This study aimed to explore the link between psychological violence (PSV) and pregnancy outcomes in terms of maternal and birth for the first time in women attending Mazandaran University of Medical Sciences (MUMS) primary health centers (PHCs) in Iran.

**Methods:** Prospective cohort of 1461 pregnant women exposed and non-exposed to PSV was followed until the pregnancy outcome. Modified Intimate Partner Violence, demographic and pregnancy outcomes questionnaires were administered face-to-face. Logistic regression analysis was down to estimate independent effects of the PSV on pregnancy outcomes.

**Results: **More than half of the women (69.9%) reported PSV during pregnancy. The differences between the two groups in reference with pregnancy complication did not reach statistical significance. Premature rapture of membrane was the only outcome that was independently associated with PSV.

**Conclusion:** PSV in pregnancy was frequent in our study. Although the lack of adverse pregnancy outcome following PSV was observed in this study, intervention is required to prevent the effect of violence on women and child health.

## Introduction

The most shameful human rights violation is violence against women (1). Published documents have been estimated the prevalence of psychological violence (PSV) during pregnancy ranged from 19.2%, 35% to 60.5% in different parts of Iran (-). If these percentages were applied to the 1,365,480 pregnant women in Iran in 2011, approximately 259,441 and 819,288 women were subjected to PSV during their pregnancies. It seems that violence might be more frequent that other medical problems during pregnancy such as preeclampsia or gestational diabetes (5).

Numerous studies have been investigated the outcomes of Intimate Partner Violence (IPV) during pregnancy, still little has been focused on PSV with positive association (6, 7). A recent study on 976 pregnant women found that the incidence of low birth weight infants was significantly increased in women who reported PSV in comparison to the no-violent group (7.6% vs. 5.1%) (8).

Early detection of PSV is important for the physical and mental well-being of mother and her child. To our knowledge, no study has been solely conducted on whether PSV against women is associated with pregnancy outcomes in Iran. This longitudinal cohort study aimed to answer two questions: a. what are the risk and protective factors for PSV against women during pregnancy, and b. what is the role of PSV on pregnancy outcomes in terms of maternal and birth outcomes?

## Materials and Methods

***Procedures***

This cohort study was conducted in the primary health centers (PHCs) of Mazandaran University of Medical Sciences (MAZUMS), the only public health centers providing prenatal care to women living in Mazandaran province in the north of Iran. Sample size was calculated based on reported prevalence of IPV in Iran and using G-power software (9). Stratified sampling method was used to selection PHCs in five parts (north, south, west, east and center) of each 16 cities. In a Poisson random method, 1500 eligible pregnant women who attended to PHCs between Februarys to September 2010 were approached. Singleton pregnant women who were not competent to give informed constant were excluded from the project. At their entry to the study, reliable socio-demographics and PSV questionnaires (Cronbach’s alpha 91%) were administered face-to-face by researchers who were familiar with the project. The women were divided into two groups; who screened positive for PSV and screened negative for PSV and then women were followed-up till outcome of pregnancy. The project was approved by Mazandaran University of Medical Sciences’ ethics committee.

***Psychological violence***

PSV was assessed using Iranian cultural adapted of World Health Organization (WHO) Domestic Violence Questionnaire that used in Iran-Tehran before and including 14 questions on PSV (4). They consist of: (a) Are you afraid of your husband? (b) Has he threatened your life? (c) Has he threatened to hurt you or anybody close to you? (d) Does he abuse you emotionally? (e) Does he use offensive language? (f) Has he verbally abused your family with or without their presence? (g) Does he disapprove of your beliefs and principles? (h) Does he curse children? (i) Does he abandon you and your children? (j) Does he keep you from going out of the home? (k) Does he keep you from going to friends or relatives, ceremonies or other places you like to go? (l) Does he keep you from getting a job? (m) Does he keep you from studying? (n) Does he keep you short in terms of money, food, and clothing? The PSV was considered “severe” if questions (a) and or (b) had a positive answer, PSV was considered “moderate” if at least five items from questions (a) to (n) were positive, and finally PSV was considered “mild” if fewer than five items were positive. This standard questionnaire was used after pretesting with a sample of 50 healthy pregnant women in PHCs with reliability of 0.78 Cronbach’s alpha.

***Pregnancy outcomes***

During the index of pregnancy, women were asked about maternal complications that included abortion (fetal loss before 20^th^ gestation week), placenta abruption (placenta separation during pregnancy), vaginal bleeding (hemorrhage in the second or third trimester) and diabetes. Birth certificates provided information on complications of delivery and birth that contained intra uterine fetal death (IUFD) (fetal loss after 20-week of gestation), intra uterine growth retardation (IUGR; birth weight was at the 10^th^ percentile or less based on published birth weights in weekly gestational age categories), premature rapture of membrane (PROM) (spontaneous ruptured of membrane four hours before beginning of delivery contraction), type of delivery (vaginal and sectarian section), pre-term delivery (live birth delivered before 37^th^ week of gestations), and low birth weight (LBW) (birth weight less than 2500 gram) (10).

***Potential confounders***

Potential confounders of the association between PSV during pregnancy and adverse maternal and birth outcomes were including age, years married, education and occupational status of the women and their husbands, family income, status of accommodation, parity and history of infertility. Iranian classification of income was used which classifies household income into three different categories: (low; less than 300 U.S. dollar, medium; 350-450 USD and high; more than 450 USD).

***Statistical analysis***

We initially explored the prevalence distribution of three groups of PSV (mild, moderate, severe) during pregnancy. Then, women were placed into two categories; one category entails women who were being PSV, and reference category involving women who were not. The prevalence of PSV in each level of maternal characteristics variables with its exact 95% confidence interval obtained from a binomial distribution was reported.

To investigate whether a relationship existed between the adverse pregnancy outcomes and PSV, simple logistic regression was used generating the corresponding odds ratios (OR) coupled with the 95% confidence intervals (CI). Then multiple logistic regression analysis was down to estimate independent effects of the PSV and kept in the model if P-value was 0.05 or less. These associations were adjusted for all confounders. Statistical Analysis software (SAS^®^), version 20.0 was used for the statistical analysis.

## Results

Data were collected through interviews with the 1461 women who were consented and followed-up the study. A small number of women (n = 39) refused to participate. Among the participants, 1,020 (69.9%) reported that they were violated psychologically by their husband during the pregnancy. The most common PSV was kept them short in terms of money, food, and clothing (88.8%).

Distribution of three groups of PSV (mild, moderate, and severe) during pregnancy is shown in table 1. More women felled in mild group of PSV (39.9%). The results of unadjusted analysis of the factors that affect on PSV are illustrated in table 2. 

**Table 1:** Prevalence severity of psychological violence during pregnancy

**Violence**	**Number (%)**
**No**	439 (30.1)
**Mild**	582 (39.9)
**Moderate**	107 (7.3)
**Severe**	331 (22.7)

**Table 2:** Prevalence of psychological violence (PSV) during pregnancy by maternal characteristics

**Characteristics** †	**Violence (n = 439)** **Number (%)**	**Non-violence (n = 1,020)** **Number (%)**	**OR (95% CI)** ‡
**Age (years)**			
** < 25**	460 (46.7)	204 (46.3)	1.01 (0.81-1.27)
** ≥ 25**	525 (53.3)	237 (53.7)	
**Gravida**			
** Primiparous**	23 (2.3)	14 (3.2)	0.72 (0.37-1.42)
** Multipara**	962 (97.7)	426 (96.8)	
**Educational status**			
** Illiterate and primary**	344 (34.9)	167 (37.9)	0.88 (069-1.11)
** Secondary and over**	641 (65.1)	274 (62.1)	
**Occupational status**			
** Housekeeper**	815 (84.9)	372 (85.1)	0.93 (0.67-1.29)
** Employed**	145 (15.1)	62 (14.3)	
**Age of husband**			
** <30**	546 (55.4)	247 (56)	0.97 (0.77-1.22)
** ≥30**	439 (44.6)	194 (44)	
**Husband's educational status**			
** Illiterate and primary**	369 (37.5)	152 (34.5)	1.13 (0.9-1.43)
** Secondary and over**	615 (62.5)	288 (65.5)	
**Husband occupational status**			
** Governmental job**	805 (81.7)	372 (84.5)	0.81 (0.6-1.10)
** Non- governmental job**	180 (18.3)	68 (15.5)	
**Polygamy**			
** Yes**	59 (6)	33 (7.5)	1.26 (0.81-1.97)
** No**	926 (94)	408 (92.5)	
**Duration of marriage (years)**			
** < 5**	611 (62)	265 (60.2)	1.07 (0.85-1.35)
** ≥ 5**	374 (38)	175 (39.8)	
**Having relative kinship with husband**			
** Yes**	133 (13.6)	73 (16.7)	0.78 (0.57-1.06)
** No**	848 (86.4)	363 (83.3)	
**Housing**			
** Own house**	412 (41.8)	202 (45.8)	1.17 (0.93-1.47)
** Renting**	573 (58.2)	239 (54.2)	
**Total household income (monthly)**			
** High (more than 450 USD; U.S. dollar)**	223 (22.7)	97 (22)	1.03 (79-1.36)
** Low (350 USD and less)**	760 (77.3)	343 (78)	

† There was no significant difference between two groups;

‡ Result from chi-square test

The characteristics of women were same in violated and nonviolent groups. Table 3 shows the association between PSV during index of pregnancy and pregnancy outcomes after adjusting. PSV had no statistically significant impact on pregnancy outcomes except PROM. There was a statistical significant difference between the violated and nonviolent women in terms of PROM [OR: 0.65 (CI: 0.43-0.97)].

**Table 3:** Association between pregnancy outcomes and psychological violence during pregnancy (adjusted analysis; n = 1,469)

**Outcome **	**OR (95% CI)**	**P-value**
**Abortion**	0.93 (0.55-1.55)	0.78
**Diabetes**	1.41 (0.63-3.15)	0.40
**BP** †	3.6 (0.44-28.8)	0.22
**Hemorrhage**	1.12 (0.49-2.56)	0.78
**Placenta abruption**	0.62 (0.27-1.41)	0.25
**Preeclampsia**	0.99 (0.59-1.64)	0.97
**Term vs. pre-term**	0.84 (0.61-1.17)	0.32
**Vaginal delivery vs. C/S** ‡	1.25 (0.97-1.61)	0.08
**PROM** §	0.65 (0.43-0.97)	0.03
**Low Birth Weight**	0.71 (0.5-1.01)	0.059

† Blood pleasure;

‡ Cesarean delivery

§ Premature rapture of membrane

## Discussion

To our knowledge, this is the first cohort study to investigate the impact of PSV solely on pregnancy outcomes in Iran. It is not easy to measure PSV because of cultural sanctioning, reluctant women to reveal it and no standardized definition of PSV (11, 12).

We found that PSV was common (69.9%) among pregnant women attending PHCs. This is comparable to the findings of previous studies conducted in Iran and indicated that PSV is more common than physical violence (60.5% and 57% vs. 14.6% and 5.5%) (4, 13). A population-based study in Hong Kong found around three-fourths of the violence during pregnancy (216 of 296 violated women) was psychological only (7). It is possible that the psychological violence could be a forerunner of other forms of violence. During pregnancy staying in touch frequently with the health service professionals provides an appropriate chance to detect who are at risk. Given the offer that progression from PSV to other forms of violence takes a short time, identifying those at risk and carry out the relevant interventions is noticeable. Further studies are needed to determine if violence is predominantly psychological in nature especially in the traditional nations.

The lack of adverse pregnancy outcome following PSV was observed in our study. The only similar findings were observed in a cross-sectional study in Hong Kong by Tiwari et al. (7). The authors found no association between PSV and LBW, pre-term birth, type of delivery, low Apgar score and admission the neonate to the hospital.

The research findings on the potential association between PSV solely and pregnancy outcomes are rare and inconclusive. Many researches explored this association in combination of all types of violence (physical, psychological and/or sexual). Zareen et al. found no statistical association between all types of violence and maternal and perinatal complications (14). This was in consistent with the findings of Covington et al., where no increase in LBW and pre-term birth was found in women who subjected to violence (15).

Certainly, the experience of violence during pregnancy has many adverse outcomes including LBW, preterm birth and premature rapture of membrane (PROM) (2, 13, 16, 17). In agreement with the above reports, positive association was found between violence and PROM in the present study. Many factors may explain this association including direct physical and health effects with mental health factors as well (12). Adverse effects are not restricted to women who subject only physical violence, as even PSV has been associated to difficult pregnancy outcomes. Women who are subjected to violence during pregnancy may experience higher rate of sexually transmitted disease (STDs) (12) that expose them at increased risk for PROM (10).

Although the present study found the lack of adverse effect of PSV on pregnancy outcomes, the psychological anger between partners can have negative effect on all family members in particular on infants (7). Thus, screening for PSV during pregnancy is needed and intervention is considered necessary not only to protect the women from adverse effects of violence, but also reduce the harm on the children. Moreover, health care providers could be able to provide limited counseling to motivational interviewing or increasing women’s self-esteem and a sense of inner control. More comprehensive research is needed to more complete understanding of IPV problem and to find appropriate solutions.

As the women participated voluntary in this study, selection bias may be occurred. The other limitation of the study is that episode of violence prior the pregnancy and following the interview may be missed. Furthermore, this study failed to provide support to the women who were subjected to PSV. 

## Notes

**Declaration of interest:** None.
